# Diagnostically Challenging Case of Renal Artery Stenosis in a Pediatric Patient

**DOI:** 10.7759/cureus.6538

**Published:** 2020-01-02

**Authors:** Osama Safdar, Fahad Alaifan, Sonds Alshammakh, Muatasaim Hakami, Daniyah F Alghaithi

**Affiliations:** 1 Pediatrics, King Abdulaziz University, Jeddah, SAU; 2 Surgery, King Abdulaziz University Hospital, Jeddah, SAU

**Keywords:** renal artery, stenosis, hypertension, blood pressure, pediatric, para, percutaneous transluminal angioplasty

## Abstract

In this study we report a diagnostically challenging case of bilateral renal artery stenosis (RAS) in a nine-year-old boy presenting as uncontrolled hypertension (HTN). The objective of this clinical case report is to draw attention to the unlikely combination of a classical clinical presentation with several normal investigations.

This is a nine-year-old boy, known to have uncontrolled HTN, later diagnosed as a case of bilateral RAS. This patient underwent several imaging studies including: renal Doppler ultrasonography (US), diethylenetriamine pentaacetic acid (DTPA) scan, and mercaptuacetyltriglycine (MAG3) renal scintigraphy with captopril challenge, with the aim of determining the cause of his uncontrolled HTN. The previously mentioned imaging procedures were carried out at two different medical centers over an extended period of time; however, the images failed to show any anatomical abnormalities to explain his clinical presentation. Due to this unexpected result the primary team in charge of this case opted for a percutaneous transluminal angioplasty, as a diagnostic and therapeutic approach, by which the final diagnosis of bilateral RAS was concluded. During follow-up the patient was asymptomatic in all outpatient clinic appointments.

Even though several initial investigations failed to indicate RAS, clinicians may pursue further investigations in cases of classical clinical presentation of uncontrolled HTN in pediatric population so as to not miss any diagnostically challenging cases.

## Introduction

Pediatric hypertension (HTN) is a common condition. Renal artery stenosis (RAS) is considered as a common cause of secondary pediatric HTN and accounts for 5%-25% of the hypertensive children [1]. RAS presents with an elevated blood pressure (BP) and can be confirmed by angiography; however, it can sometimes be diagnostically challenging [2]. In this study, we report a diagnostically challenging case of a nine-year-old boy with severe HTN due to RAS.

## Case presentation

This study was approved by the ethics committee of King Abdulaziz University Hospital (KAUH). A nine-year-old boy presented with oliguria to the ED of KAUH, Jeddah, Saudi Arabia. He was admitted for evaluation and management of his uncontrolled HTN. Prior to presentation, he was on three medications, namely amlodipine, labetalol, and hydralazine. Seven months before his presentation to KAUH, he had multiple admissions due to uncontrolled HTN and he had been investigated at the New Castel General Hospital, UK, where he had undergone a renal Doppler and magnetic resonance angiography (MRA) of the aorta and the renal arteries. The imaging modalities revealed that the aorta and the renal vessels appeared normal, with a single renal artery present on each side. The anatomy of the renal veins, kidneys, and bladder was normal. No evidence of RAS, fibromuscular dysplasia, or thrombosis was seen. 

Upon admission to KAUH, further investigations and subsequent management of his uncontrolled HTN were undertaken. On admission, his BP was 147/88 mmHg, while chest and cardiac examinations revealed no abnormalities or edema. Neurological examination revealed a normal tone, power, and reflexes, while an ophthalmic examination revealed mild disc swelling. His urine output was 2.2 mL/kg/h. He had no predisposing factors for RAS such as family history or neurofibromatosis. .

On the admission day, he was administered with a starting dose of 20 mg hydralazine and was regularly on amlodipine (5 mg BID) and labetalol (50 mg BID). Furthermore, his BP was monitored hourly; two high BP readings within 30 min garnered administration of nifedipine. If his BP remained uncontrolled, i.e. if he received two nifedipine doses within 12 h, then we planned to increase the hydralazine dosage. Consequently, he received three nifedipine doses on the admission day and only one dose the next day as his BP readings improved. 

Laboratory blood tests revealed normal hemoglobin (14 g/dL) and platelet levels (261,000 per mL/L). The level of complement C4 was low (0.18), while the C3 level was normal (0.95). His blood creatinine level was 46 mmol/L and the blood urea level was 4.8 mg/dL. Plasma renin and aldosterone levels were normal. The alkaline phosphatase level was high at 228 IU/L (normal = 40-150 IU/L). The antinuclear antibody test was negative. Urine analysis revealed clear urine with no evidence of infection. The microalbumin-to-creatinine ratio was normal (22.312 mg/dL), while the urine creatinine level was low (2080 mg/24 h).

To determine the cause of uncontrolled HTN, he underwent several imaging procedures including renal Doppler ultrasonography (US), diethylenetriaminepentaacetic acid (DTPA) scan, and mercaptuacetyltriglycine (MAG3) renal scintigraphy with captopril challenge. 

Renal Doppler US revealed that the right and left renal artery resistive indices were 0.7 and 0.5, respectively, with a peak systolic velocity of 59.7 cm/s for both. Both kidneys were average in size and echogenicity, with a preserved cortical medullary differentiation and no evidence of hydronephrosis.The right kidney measured 8 cm x 3 cm, with a cortical thickness of 0.5 cm and the left kidney measured 8 cm x 3.6 cm, with a cortical thickness of 0.5 cm.

The DTPA revealed a prompt flow, function, drainage, and good response to the lasix challenge for both kidneys, with no evidence of obstruction. The relative contribution was quantified as 44% for the left kidney and 56% for the right kidney. 

The MAG3 renal scintigraphy with captopril challenge was performed with an intravenous injection of Tc-99m MAG3, and a dynamic scan was acquired for 30 min. About 60 min prior to the study, 12 mg of captopril was injected. A postvoid image was also acquired. The findings were compared to his baseline scan. Both kidneys were normal in size, with prompt flow, function, and drainage. No evidence of cortical retention was observed and no significant change was noted when compared to the baseline scan. The relative contribution was quantified as 46% for the left kidney and 54% for the right kidney.

**Figure 1:**
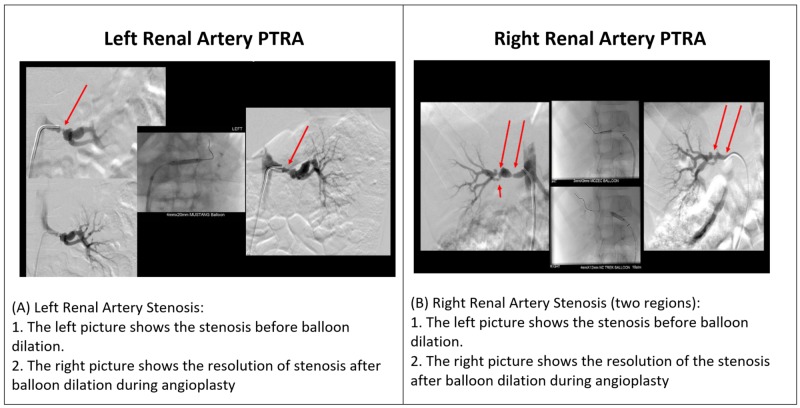
The findings of PTRA: (A) and (B) show the stenotic regions in the right and left renal arteries before and after balloon dilatation. PTRA, percutaneous transluminal renal angioplasty

Due to the fact that the imaging procedures failed to detect any functional or structural abnormalities, he was transferred to the King Faisal Specialist Hospital in Jeddah, Saudi Arabia, for a percutaneous transluminal renal angioplasty (PTRA), a diagnostic and therapeutic procedure. It revealed severe stenosis in two regions of the right renal artery and one region of the left renal artery, as shown in Figure 1. The PTRA findings are represented in Table 1.

After he underwent PTRA, his medications were discontinued. His BP readings were normal during follow-up visits for three months.

**Table 1:** Pre- and post-PTRA results. PTRA, percutaneous transluminal renal angioplasty

		Pre-PTRA (mmHg)	Post-PTRA (mmHg)
Left renal artery	Left renal artery mean blood pressure	58	75
	Aorta mean blood pressure	78	79
	Gradient blood pressure	20	4
Right renal artery	Right renal artery mean blood pressure	35	90
	Aorta mean blood pressure	102	92
	Gradient blood pressure	67	2

## Discussion

Hypertension presents in more than 3% of pediatric individuals [3]. Among these, secondary HTN is more common than primary HTN, and can often cause significant morbidity if its etiology is undetermined and untreated [4]. Among secondary HTN patients, 10% have a renovascular cause, which is often caused by RAS [5-7]. Patients with renovascular HTN could be asymptomatic or could present with nonspecific symptoms related to end-organ effects [8].

In adults, RAS mostly occurs due to aortic atherosclerosis. In pediatric patients, isolated branch RAS tends to be more common [8]. A study done on 21 pediatric RAS patients reported that 50% of the detected lesions were of first-order isolated renal branch artery stenosis. Stanley and Fry also reported that eight of their 24 pediatric patients had isolated extrarenal branch artery stenosis with no associated systemic diseases [9]. 

Renal artery stenosis can be diagnostically challenging [8]. Several imaging modalities can be used to diagnose RAS. These include Doppler US, conventional CT, renal scintigraphy, MRA, and CT angiography (CTA) [10]. 

There are various opinions regarding the optimal diagnostic strategy, as the patient’s age, need for sedation, likelihood of renovascular disease, and the use of contrast and radiation must be taken into consideration [11]. A recent study reported low specificity and sensitivity of captopril renography in children [12]. CTA and MRA can be used, with variable detection rates [13-14]. Conventional contrast angiography (CCA) is considered the gold standard modality. However, it is regarded as an invasive procedure, as it requires contrast injection and carries a radiation burden. In cases where diagnosis is difficult by less invasive modalities, PTRA can be used. In our case, no single imagining modality was able to diagnose RAS, with the exception of PTRA, which was used as the last resort.

The options for treating RAS are controversial and the choice depends upon its severity and location [7]; even then, the initial treatment is usually pharmacological [8]. Renal artery revascularization is another treatment option and is considered when the patients are suffering from uncontrolled HTN or progressive renal dysfunction, which can be established through PTRA [15-16].

A previous report on two pediatric cases illustrated the difficulty in diagnosing branch RAS, even by CCA. Stenosis in the first case was initially excluded based on the patient’s normal CTA findings and the high-normal value of p-renin. However, stenosis was diagnosed after a positive captopril renography scan, which revealed significant changes after being repeated without captopril. It was confirmed and treated by renal angiography [8]. In the second case, branch artery stenosis was diagnosed on the basis of Doppler US and captopril renography findings. Furthermore, renal angiography confirmed the diagnosis by showing a secondary branch of the left RAS. The patient was treated by PTRA [8].

The diagnosis of our patient was challenging because he underwent multiple investigations, all of which failed in detecting RAS. PTRA was the diagnostic and therapeutic modality of choice. Table 2 summarizes previous pediatric case reports on RAS, with their imaging modality findings and their diagnosis and management. 

**Table 2:** Summarized pediatrics case reports on RAS from existing literature. RAS, renal artery stenosis; BP, blood pressure

Ref.	Year	Age/Sex	Diagnosis	Treatment	Outcome
[8]	2017	14-year-old female	1. Doppler ultrasound of the renal arteries: stenosis in the right side renal artery. 2. Captopril renography: abnormal right renogram. 3. Renal angiography: severe stenosis of the right renal artery caudal branch due to fibromuscular dysplasia	PTRA	Three months after treatment, his 24-h BP improved to 117/61 mmHg without medications.
[8]	2017	22-year-old female. Hypertension was incidentally discovered at the age of 13 years	Renal angiography detected tight stenosis in the secondary branch of the left renal artery caused by fibromuscular dysplasia. 1. Initial diagnosis by Doppler US and captopril renography and renal vein catheterization findings: left renal artery stenosis. 2. Angiography revealed stenosis in the secondary branch of left renal artery due to fibromuscular dysplasia.	PTRA	The high BP was ameliorated, and captopril renography findings were normalized.

## Conclusions

The diagnosis of RAS is challenging and requires a variety of investigations. However, no single imaging modality can diagnose RAS and it can be missed in some cases. In our case, PTRA was the only imaging modality that detected RAS.
